# Taming the pandemic? The importance of homemade plant-based foods and beverages as community responses to COVID-19

**DOI:** 10.1186/s13002-020-00426-9

**Published:** 2020-12-09

**Authors:** Andrea Pieroni, Ina Vandebroek, Julia Prakofjewa, Rainer W. Bussmann, Narel Y. Paniagua-Zambrana, Alfred Maroyi, Luisa Torri, Dauro M. Zocchi, Ashley T. K. Dam, Shujaul M. Khan, Habib Ahmad, Yeter Yeşil, Ryan Huish, Manuel Pardo-de-Santayana, Andrei Mocan, Xuebo Hu, Odara Boscolo, Renata Sõukand

**Affiliations:** 1 University of Gastronomic Sciences, Bra/Pollenzo, Italy; 2 Medical Analysis Department, Faculty of Science, Tishk International University, Erbil, Kurdistan Region Iraq; 3 Institute of Economic Botany, The New York Botanical Garden, New York, USA; 4 The Forest School at the Yale School of the Environment, New Haven, CT USA; 5 PhD Program in Biology, The Graduate Center of the City University of New York (CUNY), New York, USA; 6 Ecology, Evolution and Environmental Biology (E3B) Department, Columbia University, New York, USA; 7 Department of Environmental Sciences, Informatics and Statistics, Ca’ Foscari University of Venice, Venice, Italy; 8 Department of Ethnobotany, Institute of Botany and Bakuriani Alpine Botanical Garden, Ilia State University, Tbilisi, Georgia; 9 National Herbarium of Bolivia, Institute of Ecology, Universidad Mayor de San Andrés (UMSA), La Paz, Bolivia; 10 Department of Botany, University of Fort Hare, Alice, South Africa; 11 Department of Plant Sciences, Quaid-i-Azam University, Islamabad, Pakistan; 12 Department of Genetics, Hazara University, Mansehra, Pakistan; 13 Department of Pharmaceutical Botany, Faculty of Pharmacy, Istanbul University, Istanbul, Turkey; 14 Department of Natural Sciences, The University of Virginia’s College at Wise, Wise, VA USA; 15 Department of Biology, Autonomous University of Madrid, Madrid, Spain; 16 Centro de Investigación en Biodiversidad y Cambio Global (CIBC-UAM), Madrid, Spain; 17 Department of Pharmaceutical Botany, Iuliu Hațieganu University of Medicine and Pharmacy, Cluj-Napoca, Romania; 18 Laboratory of Chromatography, Institute of Advanced Horticulture Research of Transylvania, University of Agricultural Sciences and Veterinary Medicine, Cluj-Napoca, Romania; 19 Institute for Medicinal Plants, College of Plant Science and Technology, Huazhong Agricultural University, Wuhan, China; 20 Laboratory of Economic Botany and Ethnobotany, Department of Biology, Fluminense Federal University, Rio de Janeiro, Brazil

**Keywords:** Ethnobotany, Ethnomedicine, Food medicines, Ethnogastronomy, Healthy food, COVID-19, Food plants, Herbal teas, Community health

## Abstract

Household responses to COVID-19 in different corners of the world represent the primary health care that communities have relied on for preventing and mitigating symptoms. During a very complex and confusing time, in which public health services in multiple countries have been completely overwhelmed, and in some cases even collapsed, these first-line household responses have been quintessential for building physical, mental, and social resilience, and for improving individual and community health. This editorial discusses the outcomes of a rapid-response preliminary survey during the first phase of the pandemic among social and community contacts in five metropolises heavily affected by the COVID-19 health crisis (Wuhan, Milan, Madrid, New York, and Rio de Janeiro), and in twelve rural areas or countries initially less affected by the pandemic (Appalachia, Jamaica, Bolivia, Romania, Belarus, Lithuania, Poland, Georgia, Turkey, Pakistan, Cambodia, and South Africa). We summarized our perspectives as 17 case studies, observing that people have relied primarily on teas and spices (“food-medicines”) and that there exist clear international plant favorites, popularized by various new media. Urban diasporas and rural households seem to have repurposed homemade plant-based remedies that they use in normal times for treating the flu and other respiratory symptoms or that they simply consider healthy foods. The most remarkable shift in many areas has been the increased consumption of ginger and garlic, followed by onion, turmeric, and lemon. Our preliminary inventory of food medicines serves as a baseline for future systematic ethnobotanical studies and aims to inspire in-depth research on how use patterns of plant-based foods and beverages, both “traditional” and “new”, are changing during and after the COVID-19 pandemic. Our reflections in this editorial call attention to the importance of ethnobiology, ethnomedicine, and ethnogastronomy research into domestic health care strategies for improving community health.

**Supplementary Information:**

**Supplementary information** accompanies this paper at 10.1186/s13002-020-00426-9.

## “Edible medicines”

In many cultures, homemade plant-based beverages and foods represent a crucial part of the folk medical repertoires on which communities rely for treating minor, infectious, and chronic diseases [1]. These folk remedies are embedded within a broader framework of Traditional/Local Environmental Knowledge (TEK/LEK) systems, which are defined as coevolving conglomerates of knowledge, beliefs, and practices that are transmitted mainly, albeit not exclusively, verbally [2]. Moreover, Nina Etkin introduced the pioneering concept of “edible medicines” [3] to describe those domestic foods that are used to improve the general health of community members, i.e., preparations that adhere to the aesthetics of taste, appearance, and smell (and thus producing pleasure) but simultaneously function as remedies. In spite of a huge interest in folk remedies by ethnopharmacology over five decades [4], robust evaluations of common folk edible medicines retained within local communities are still lacking, since pharmacological research has been primarily directed at plant remedies traditionally prescribed or recommended by healers and other alternative medicine professionals and has neglected or overlooked domestic food medicines.

There exists an emerging literature consisting of reviews and commentaries on promising dietary and herbal remedies for prevention and treatment of COVID-19 [5–8], but the relevance of this information compared to the plant remedies that local communities are actually using remains unclear, and participatory approaches in conducting ethnobiological field studies during the current pandemic are lacking [9]. In this editorial, we aim to fill that gap by providing perspectives from different countries as case studies, as well as a preliminary (baseline) inventory of plant remedies recorded during the first phase of the pandemic. The provision of health care via food at the household level is a crucial issue for public health, since homemade foods represent the primary health care remedies that communities rely on in times of crisis.

The two main objectives of the rapid preliminary inquiry presented in this editorial consisted of (1) documenting popular plant *food medicines* (homemade, plant-based healing foods, and beverages) and (2) to impressionistically assess changes in their consumption or intake, during the COVID-19 pandemic.

## A preliminary survey

During the first phase of the pandemic (December 2019–August 2020), the authors used rapid-response preliminary inquiry and participant observation, conducting at least 10 interviews (mainly through remote and online inquiry) based on convenience sampling (reaching out to their social contacts, including those living in local communities) and often following the snowball method in the area in which they live, or where they normally conduct field studies. Five large urban (metropolitan) areas that were heavily affected during the first phase of the COVID-19 health crisis (Wuhan, Milan, Madrid, New York, and Rio de Janeiro), and twelve rural areas or other countries that were initially less affected by the pandemic (Appalachia, Jamaica, Bolivia, Romania,Belarus, Lithuania, Poland, Georgia, Turkey, Pakistan, Cambodia, and South Africa), were included in this study. Study participants were asked to describe (1) homemade plant foods and beverages that they had used during COVID-19, for preventing infection and/or for treating symptoms, and (2) changes in the consumption of specific plant foods in response to COVID-19. Further details on the methodology are presented in Additional file 1. The inquiry represented a preliminary appraisal that will inform more systematic (broader and deeper) future research. In addition, in order to better understand the role of social media, the data of the Polish, Lithuanian, and Belarusian case studies was obtained from discussion threads on social media and Internet news portals (Additional file 2). Since the number of people (or Internet posts) in our sample varied by country, city, or region, we introduced the relative importance (RI) value (calculated as the percentage of people or internet posts mentioning a specific plant in every case study) and tallied RI values to quantify the cross-regional relative importance of individual plant taxa.

Botanical identification of plants was based on the authors’ expertise with the correspondence between local and scientific names in their regions, and scientific names follow The Plant List [10], while family assignments follow the Angiosperm Phylogeny Group (APG) IV [11]. Organisms other than plants were mentioned by some participants (including fungi, algae, and animal products), but we mostly report on plants.

The motivation for this editorial was to obtain a rapid first impression of community responses around the world, which represents valuable baseline data for future studies and continued in-depth research. This is especially important since TEK/LEK systems are known to actively respond to epidemiological trends, and therefore, plant pharmacopoeias are likely to change as the pandemic continues to unfold. A weakness of this approach is the lack of comprehensive field work, which represents the gold standard in ethnobotany, including the ability to build community rapport about the topic of research, conduct in-person interviews, pre-test survey questionnaires, and collect voucher specimens for botanical identification. Although institutional ethics review was not obtained for this initiative because of time constraints (rapid appraisal), the upheaval of administrations around the world, and barriers to conduct field work, the authors adhered to the ethics guidelines set out by their professional societies and followed Free, Prior, and Informed Consent (FPIC). The 17 case studies were summarized briefly, which limited the depth of information. However, all recorded plant taxa are provided in Additional file 3. The strength of this study is that insights into community-based responses to this global health crisis are urgently needed, underexplored, and underrepresented in the literature. The diversity of case studies represented in this paper shows a rich overview of how communities across the globe are actively managing their health in real time and coping during this crisis, using plants and their TEK/LEK systems.

## Community responses

The following paragraphs represent 17 case studies that briefly summarize perspectives of community responses to COVID-19 with homemade plant foods, beverages, and teas around the world. The name of each region, city, or country is followed by the initials of the author in brackets. Scientific names of all reported plant species, in addition to the ones reported in the text, can be found in Additional file 3.

## Wuhan, China (XH)

Wuhan was the endemic center of COVID-19. At the end of December 2019, the local health agency released information to the media that suspected severe acute respiratory syndrome (SARS) was observed in the city. Because many Chinese people remember the 2002 SARS outbreak, some over-the-counter (OTC) Traditional Chinese Medicine (TCM) ran quickly out of stock in drug stores. This included the famous extraction of banlangen roots (*Isatis tinctoria* L. or *Strobilanthes cusia* (Nees) Kuntze), a first-response remedy to virus infections for many Chinese people. Once banlangen and similar OTCs became unavailable, many people tried other foods and drinks, including hot tea (*Camellia sinensis* (L.) Kuntze). The theory of TCM prescribes heat to fight viruses, so people who did not like tea drank hot water instead. Other dietary interventions included soup of ginger and pear (*Pyrus* spp.), pear with rock sugar, ginger with coca-cola, or soup made with beef, lamb, steak, or fish. Many people also considered porridge important, made with yam (*Dioscorea oppositifolia* L.), longan (*Dimocarpus longan* Lour.), date (*Ziziphus jujuba* Mill.), or wolfberry (*Lycium barbarum* L.). The national administration of TCM and its branches released different versions of TCM formulas for treating COVID-19. By law, these TCM formulas should be prescribed by doctors. Because COVID-19 treatment is limited to specialized free hospitals in Wuhan, these TCMs were provided to patients through government-contracted companies. At the end of January 2020, Hubei Province, of which Wuhan is the capital, released two specific TCM formulas for preventing COVID-19. None of our interview respondents had prepared such tea formulas themselves; however, a Wuhan company claimed to have made over 3 million bags of the drink, and sent it to city hospitals and communities, based on a local news report.

## Milan, Italy (LT/DMZ)

During the lockdown, there was a regular consumption of foods associated with perceived beneficial effects, such as fruits and vegetables, brown rice, spelt (*Triticum spelta* L.), legumes, and spices, including cinnamon and ginger, hemp, and extra-virgin olive oils, as well as almond milk. At the same time, an increase in the consumption of some specific fresh fruits (especially citrus fruits and strawberries), broccoli, leafy vegetables (e.g., *Spinacia oleracea* L.), onions, and bell peppers, as well as hot herbal teas, was observed. Moreover, we noticed the introduction of some superfoods into the diet, such as spirulina (*Arthrospira platensis* Gomont), Manuka honey, matcha tea (*Camellia sinensis*), and echinacea (*Echinacea* sp.), particularly among the youngest community members. It seems that changes in food consumption habits adopted to face COVID-19 were mainly based on suggestions offered by professionals (e.g., pharmacists, herbalists, and social workers in the public health service) and also by information available on the web or received via WhatsApp.

## Madrid, Spain (MPS)

As in several metropolises, the pandemic has had grave consequences in Madrid, with nearly everybody having close friends and relatives that were severely or mildly affected, many of them treated in hospitals but most at home. Doctors made their diagnosis over the phone and mainly recommended paracetamol. It seems that the pandemic reinforced trends in the consumption of herbal teas and healthy plant-based foods. Most people interviewed (10/13) had taken home remedies to prevent or cure coronavirus symptoms, primarily those that they normally use when they are suffering from cough or throat problems, including homemade orange and lemon juice, tea of ginger and thyme (*Thymus vulgaris* L.), and sucking on dried ginger. Remedies for enhancing immune defenses, such as garlic, lemon juice, apple vinegar, and herbal teas (e.g., green tea, nettle, dandelion) were also taken. Loss of appetite was commonly reported, and improving the quality of food was mentioned as very important. People also increased their intake of healthy foods, such as fruits, mixed and colorful salads, other vegetables, and legumes. Most people stated that they had learned these remedies from close relatives, as well as from teachers, books, and magazines. Health authorities promoted healthy diets rich in fruits, legumes, and vegetables, using olive oil and consuming nuts daily. Such diets, as well as those that increase defenses and are rich in antioxidants (e.g., lemon, oregano, salvia, licorice), were encouraged by the Internet and WhatsApp messages. However, newspapers and other websites also discouraged treatments that they considered fake news (e.g., claims that drinking hot beverages or lemon juice kills the virus).

## New York City (Caribbean communities), USA (IV)

Half of the top ten countries of origin of New York City’s (NYC) immigrant population are Caribbean. Data from NYC’s Health Department tells the grim tale of COVID-19 for this population, by showing that NYC neighborhoods with the highest percentage of COVID-positive residents included heavily populated Caribbean sections of the Bronx, Brooklyn, and Queens. Around mid-April 2020, 1 month into NYC’s closure, a former research assistant reached out via email with the observation that many people in her community, namely New Yorkers hailing from the Dominican Republic, had resorted to using thyme for self-care in response to this new virus. Ingested as an infusion, used culinarily, or applied as a body oil, information about the use of thyme for COVID-19 was readily communicated among family and friends through WhatsApp. During the first peak of the pandemic (spring 2020), a *botánica* (shop selling Caribbean remedies for health and wellbeing) in the Bronx reported selling much greater volumes online of eucalyptus, chamomile, and *anamú* (*Petiveria alliacea* L.), to treat COVID-19 symptoms such as cough, fever, and trouble breathing, together with other plants that allegedly ease anxiety. In an 8-min home video shared on Facebook, a Jamaican-American woman demonstrated how to make “Corona-shots” by blending common Caribbean kitchen ingredients (turmeric, lemon, ginger, onion, garlic, cayenne pepper, apple cider vinegar, and honey). These examples show how plant knowledge has found its way to effective and rapid audio-visual transmission pathways (phone, internet, printed media) during this health crisis, fostering community resilience and agency.

## Rio de Janeiro, Brazil (OB)

The general population lacked appropriate information about COVID-19, and the negligence of the government made the situation very dangerous. In the period of confinement, it was crucial for inhabitants to envision alternatives in order to keep the mind healthy and also to strengthen their physical and emotional well-being. In addition to respiratory symptoms caused by COVID-19, locals suffered often from anxiety, depression, stress, and insomnia. We could observe that plants sought after were those most commonly used in the treatment of a regular flu. Lavender was used in the form of tea or for steam inhalations, as a decongestant and sedative; lemongrass, *erva cidreira* (*Lippia alba* (Mill.) N.E.Br. ex Britton & P.Wilson), *pitanga* (*Eugenia uniflora* L.), garlic, and ginger have been used in the form of tea and were often associated with the ubiquitous lemon. Chamomile and passion fruit were utilized against insomnia and anxiety, while *guaco* (*Mikania cordifolia* (L. f.) Willd.) was widely used in the form of syrups, as well as *inhame* (taro, *Colocasia esculenta* (L.) Schott) tubers, which were eaten (cooked or raw) to increase immune defenses.

## Rural South-Central Appalachia, USA (RH)

Participants acknowledged over 60 taxa (including plants, fungi, animals, and bacteria) used in response to the pandemic, 29 of which are native to the Appalachian region. Many of the cited uses originated with indigenous communities, while other uses were introduced later through different folkways or popular media. These uses of plants (and fungi) mainly fell into three general categories: (1) consumed to stimulate the immune system (such as native reishi mushrooms (*Ganoderma* spp.), (2) used as a specific antiviral (such as elderberry, *Sambucus* spp.), and (3) ingested for general health (vitamins, minerals, and other beneficial compounds, or for microbiome enhancement) to strengthen and protect the body during the pandemic (such as wild-collected ramps, *Allium trichoccum* Aiton). One recurring theme from participants was “going back to the traditional ways,” whether that was directly stated or implied based on the items and behaviors they cited. Some explained that they and many others who retained the “old ways” felt a deeper value and appreciation for these traditions, and people would come to them more frequently for information during the pandemic. Some of the older generation even paired with the younger to create social media platforms, online video channels, and community-organizing efforts in direct response to these increased needs and inquiries. This may exemplify the principle of “dormant ethnobotany,” whereby enduring ethnobiological traditions—like a seed bank of knowledge—experience a greater re-emergence, catalyzed by the need to sustain life, well-being, and livelihoods. This emphasizes the importance of documenting traditional ethnobiological knowledge and protecting biological and cultural diversity in responsible ways.

## Jamaica (IV)

Until early August 2020, Jamaica had less than 1000 confirmed COVID-19 infections and 13 associated fatalities, the majority occurring in urban areas (surrounding the capital and a call center in a neighboring parish), while fewer cases had been recorded in rural areas. Perhaps unsurprisingly, then, there were more preliminary reports of COVID-19 plant remedies from urban than rural Jamaicans. Rapid inquiry tallied 31 species reported by 17 Jamaicans to prevent COVID-19, of which most plants (23) were common Caribbean foods used as seasonings, vegetables, homemade fruit juices, or hot beverages (teas), the most popular being ginger, sour or Seville orange, garlic, lime, turmeric, and bissy (kola nut, *Cola acuminata* (P.Beauv.) Schott & Endl.). Cultural explanations for preventing COVID-19 infection emphasized plant remedies that were already known by Jamaicans to (1) treat the common cold, flu, or fever (known as virus remedies); (2) “boost the immune system;” (3) “fight inflammation;” (4) “warm up the body;” (5) “remove toxins;” (6) prevent blood clotting; (7) provide vitamins (especially vitamin C); or (8) plants considered natural and nutritious, including heirloom varieties, traditional landraces, and wild greens, such as gully beans (*Solanum torvum* Sw.), moringa (*Moringa oleifera* Lam.), watercress, ruderal leaf amaranths, and purslane. Claims from street and market vendors about the putative properties of natural remedies for COVID-19 may have even increased their market value, which is what allegedly tripled the price of garlic. The media quickly picked up on the increasing popularity of specific plants during the pandemic, such as vervine (*Stachytarpheta jamaicensis* (L.) Vahl and *Stachytarpheta cayennensis* (Rich.) Vahl).

## Bolivia (NPZ)

In the countryside, the Andean population widely believes that a traditional diet based on *chuño* (freeze dried potato) and *kaya* (freeze dried *Oxalis tuberosa* Molina) provides immunity to SARS-CoV-2. In contrast, the population in the lowlands, where the incidence of COVID-19 is highest, does not make any links between the benefits of traditional foods and the prevention and treatment of the virus. Although there are policies that recognize the importance of traditional medicine, none include food. For the prevention of COVID-19, the Ministry for “traditional” medicine has widely promoted in the media the consumption of herbal teas or the inhalation of steam from preparations of introduced species like chamomile, cypress (*Cupressus sargentii* Jeps.), lemongrass, and eucalyptus. Teas of native species, especially *Achyrocline alata* (Kunth) DC., have also been employed. Steam mixtures of these species are being used in “disinfection chambers” at the entrances of public markets, demonstrating great ignorance of the correct application of these species for the prevention of COVID-19. The consumption of onions, garlic, citrus fruits, ginger, and turmeric to strengthen the immune system during the pandemic has recently been promoted in many social networks.

## Transylvania, Romania (AnM)

In rural Romania, the intensity of security and sanitary measures imposed due to the COVID-19 pandemic has been inversely related to the degree of isolation of rural communities. Moreover, many city inhabitants spent the COVID-19 “lockdown” in isolated rural areas. The media has had a large influence on social awareness of the pandemic’s effects, and people have started to reshape their daily habits related to domestic (plant-based) food medicines by increasing their consumption, especially of herbal teas and healthy plant-based foods perceived to counteract the respiratory symptoms of flu or to enhance immunity. Different species of wild thyme and mint, as well as the buds of European silver fir (*Abies alba* Mill.) or pine (*Pinus* spp.) have been used in teas, syrups, or as extracts mixed with honey or propolis tinctures to treat or prevent common respiratory symptoms of any flu. Furthermore, sea buckthorn (*Elaeagnus rhamnoides* (L.) A.Nelson) stored in honey is usually consumed in the morning before any meal. Garlic, onion, shallot, garden parsley, stinging nettle (*Urtica dioica* L.), fat hen (*Chenopodium album* L.), sorrel (*Rumex acetosa* L.), and ramsons (*Allium ursinum* L.) are normally consumed in high amounts in spring, either raw in salads or in soups (garden parsley in juices or smoothies), and they are considered healthy foods with a role in preventing colds and enhancing immunity.

## Poland (JP)

Based on Google Trends searches, we saw a fairly high surge of interest in information about homemade remedies against the coronavirus starting around February 2020. This has declined since an initial peak in April as lockdowns have eased, but still remained popular in July and early August 2020. Around mid-February 2020, study participants started to actively create discussion threads on Facebook groups, sharing all kinds of treatments and preventive measures for COVID-19. At the same time, propaganda in social media by “COVID deniers” was quite popular in the study area as well. During the outbreak, participants shared more recipes for medicinal plants than healthy plant-based foods used in the prevention and treatment of COVID-19. This is due to the common availability of medicinal plants and because print and digital sources play a major role in transmitting ethnomedical knowledge. Plants that were commonly mentioned include licorice, black elderberry (*Sambucus nigra* L.), and nettle (*Urtica dioica* L.).

## Lithuania (JP)

Compared to Poland and Belarus, the most diverse pattern of using food as medicine was documented in Lithuania. Since the announcement of the official lockdown, Internet discussion increased about herbal remedies and medicinal foods that can be used for cough and fever, linking these symptoms with COVID-19. In pandemic news discussion forums, people mostly shared ways on how to boost the immune system. During the pandemic, professional herbalists played a key role in popularizing traditional antiviral remedies, creating an aura of therapeutic credibility around homemade plant-based foods and beverages. They actively promoted the use of plants and honeybee products for treatment and prevention of COVID-19. The most frequently reported species were garlic, nettle, and *Tropaeolum majus* L.

## Belarus (JP)

Health discussions around “miracle cures” and prevention of the coronavirus through medicinal foods have been drawing on traditional narratives of danger and local ethnomedical knowledge. In Belarus, no official lockdown was announced. State-owned media provided scarce news coverage and limited promotion of plant food medicines compared to Poland and Lithuania. Yet, the effectiveness of promoting lemon and ginger in official media discourse increased their demand and price in the spring of 2020. On the other hand, local community members preferred the remedies of the Soviet pharmacopoeia in this crisis situation. Therefore, based on our observations, people were forced to return to their former experience of preventing acute respiratory infections with homemade plant-based foods and beverages. The most important use-subcategories reported were raw vegetables, berry compotes, alcoholic beverages, honeybee products, and fungi, especially chaga mushroom (*Inonotus obliquus* (Fr.) Pilát), which is often perceived as a plant in folk categorization.

## Georgia (Caucasus) (RB)

Although a cradle for crop wild relatives, the shift from ancient cultivars to modern high-yielding crops such as maize and potato, and the educational indoctrination of the population that eroded traditional knowledge under Soviet occupation, have profoundly changed the concept of local medicine in Georgia. Self-medication during COVID-19 has been largely based on Central European herbal remedies that had previously been promoted during Soviet times. Food plant use in response to SARS-CoV-2 was restricted to syrups and teas prepared from well-known species like onion, chili, chamomile, radish, coltsfoot (*Tussilago farfara* L.), Koch’s pine (*Pinus kochiana* Klotzsch ex K. Koch), rose, and elecampane (*Inula helenium* L.). In rare cases, teas (regarded as food) made from wormwood (*Artemisia absinthium* L.), *Pyrethrum parthenifolium* Willd., *Salvia verticillata* L., *Rhododendron caucasicum* Pall., *Rubus* spp., *Sambucus ebulus* L., and *Viburnum opulus* L. have been used to alleviate flu symptoms and fever.

## Eastern Turkey (YY)

Using edible plants as a means of healing goes hand in hand with the belief that healing comes from nature and that what is wild is healthier. In Eastern Turkey, some edible plants, such as *Anchusa* spp., and *Allium* spp., which are collected in the spring, are consumed both as a food and for medicinal purposes (to increase immune defenses). In particular, local people drink herbal tea prepared with the fruits of *Rosa canina* L. to treat or prevent common respiratory symptoms and increase immune defenses, and drink a cold beverage made with the roots of licorice as an immune stimulant. The use of linden (*Tilia* spp.) and Malvaceae (*Alcea* and *Althaea* spp.) was also recorded in cases of possible COVID-19 symptoms (shortness of breath and cough). Our observations show that mint, oregano, thyme, and *Thymbra* spp., which are generally consumed as spices in the study area, have also been used in herbal teas to treat or prevent common respiratory symptoms of any flu, and that mint species have been used as incense as well. In addition, *Salvia multicaulis* Vahl was recorded as an herbal tea for respiratory diseases. Finally, the fruits of *Celtis tournefortii* Lam. were fried, crushed and powdered, and prepared in a mixture with honey or molasses, which is eaten to boost the immune system.

## Khyber Pakhtunkhwa, Pakistan (SMK/HA)

Pashtuns in this area live in close contact with nature and have thus utilized plants and other natural products during the COVID-19 pandemic. It was experienced, observed, and heard that not only the use of certain vegetables and fruits considered beneficial increased significantly during the pandemic, i.e., lemon, banana, ginger, garlic, onion, cucumber, apple, peach, cherry, plum, apricot, and date, but that their demand and prices increased in the market as well. Certain spices, like turmeric, nigella, senna (*Cassia angustifolia* Vahl), and licorice, went out of stock in certain markets for a while, despite continuous supply during lockdowns. People believe that some of these are immune boosters, while other plants are considered bronchodilators. A few individuals reported using an interesting mixture of powdered almonds, walnuts, hazelnuts, nigella, poppy seeds, and the resin of *Dalbergia sissoo* DC., mixed with brown sugar. Some interviewees also reported drinking various herbal teas, especially one prepared with the fruit epicarp of poppy (*Papaver somniferum* L.) alone, or in combination with ginger, before going to bed.

## North Cambodia (ATKD)

Cambodians have been vigilant in their efforts to minimize potential virus transmission, despite having low numbers of COVID-19 cases nation-wide in comparison to other Southeast Asian nations. As of the beginning of August 2020, 243 cases were recorded and no people have died. Eighty-nine percent of COVID-19 cases were foreign nationals visiting Cambodia or Khmer nationals returning from abroad. Drawing from various sources such as Facebook, YouTube, and word-of-mouth from peers, Cambodians have been re-orienting their diets to include more diverse foods typically designated as immune-boosting and having a humoral “hot” quality within Traditional Khmer Medicine. According to various interviewees from rural villages in Siem Reap Province, foods which have been circulated as a prophylaxis or treatment of COVID-19 include ginger (most common), garlic, chili (*Capsicum annuum*, “bird’s eye”), salt, lemon, hard-boiled eggs, dried salted fish, and rice porridge. While some of these foods have been incorporated into other dishes, many of them have been consumed as teas. High interest in ginger during the pandemic has caused spikes in its pricing at local market stands and corner shops. As one Cambodian woman explained, “it [ginger] is normally 4000 riels (1 USD), but now the price has gone up almost five-fold during the pandemic…very expensive!”.

## South-Africa (AlM)

All individuals interviewed in Alice, South Africa, perceived the importance of recreational and herbal teas, which were considered “healthy” during the COVID-19 pandemic. The majority of used plant species were prepared as recreational teas. Rooibos (*Aspalanthus linearis* (Burm.f.) R. Dahlgren) was the herbal tea mentioned by all participants, while regular tea (*Camellia sinensis* (L.) Kuntze), and tea of *Lippia javanica* (Burm.f.) Spreng., *Cyclopia* spp., *Fadogia ancylantha* Schweinf., and ginger were cited by nearly half of all interviewees. Herbal teas were regarded as an important category of hot beverage that was essential during the COVID-19 outbreak, being consumed for their sedative or stimulant effects, and also for their pleasant flavor or aroma.

## Overview of reported plant remedies

Additional file 3 provides a detailed overview of 193 plant taxa from 69 families used in the 17 selected countries, regions, or cities. Thirty-seven taxa had a cumulative relative importance (RI) equal to, or higher than, 0.5. Of these taxa, eighteen were used in at least three regions. Combining these two criteria (RI value and number of regions) highlights the ten most important taxa used as home-remedies during the first phase of the COVID-19 pandemic across all 17 case studies (Table 1).

**Table 1:** Popular plant taxa used in at least three case studies with a cumulative RI value higher than 1

Plant taxa	Common name (English)	# Case studies reporting	Sum of RI
*Zingiber officinale* Roscoe	Ginger	14	4.9
*Allium sativum* L.	Garlic	12	3.8
*Allium cepa* L.	Onion	9	3.7
*Curcuma longa* L.	Turmeric	7	2.9
*Citrus limon* (L.) Osbeck	Lemon	7	2.7
*Matricaria chamomilla* L.	Chamomile	5	1.8
*Camellia sinensis* (L.) Kuntze	Tea	5	1.6
*Urtica dioica* L.	Nettle	4	1.4
*Capsicum annuum* L.	Chili pepper	4	1.2
*Malus domestica* Borkh.	Apple	6	1.1

The RI (relative importance) value was calculated as the proportion of people (or Internet posts for Belarus, Lithuania, and Poland) mentioning a plant species in each case study (by country, city, or region). The cumulative RI value represents the sum over all case studies

Since the onset of the COVID-19 health crisis, many communities around the world seem to have gone back to relying on their domestic remedies while public health services collapsed, as happened, sadly, in the municipal areas of Lombardy and Milan, Madrid, and New York City.

Our preliminary assessment shows that the pandemic has widely reinforced the relevance and use of medicinal plants and healthy foods as home remedies, even in large cities, whereby local traditions and “imported” practices based on the use of “new” plant remedies can hybridize and coexist. For example, in Spain, turmeric and ginger did not exist in the traditional local pharmacopoeias, but these “imported” remedies now coexist with indigenous plants that have been locally cultivated or gathered with a long history of use, such as lemon and thyme.

The different types of domestic preparations that community members reported to prevent or treat COVID-19 are those that were formerly known to cope with the flu and other respiratory illnesses or symptoms, including the following:

- Folk remedies and foods specifically aimed at preventing illnesses;
- Folk remedies and foods for strengthening the body, a health strategy that has been described until a few decades ago as “the reconstitution effect”, or more recently in the modern narrative as “boosting the immune system”;
- Folk remedies directly used for alleviating respiratory symptoms.

## Future perspectives

Our case studies from around the world highlight the following important findings:

- Plant-based domestic teas, other beverages, and foods “of the past” (known as folk or traditional medicines) represent the initial community health response in times of uncertainty, and when public health services are under pressure;
- The consumption of certain plant foods (most notably garlic and ginger, but also onion, lemon, and turmeric) has remarkably increased in response to COVID-19;
- Some homemade remedies have been popularized via the Internet and social media, notably (but not exclusively) among younger community members. Therefore, these pathways of information dissemination, and sources of sharing of ideas at the community level, warrant further study as a platform for the targeted transmission of information supporting community health;
- Health behavior responses such as these are relevant, not only because they involve bioactive “phytochemicals,” but, more importantly, because they foster social cohesion, agency, and resilience, or a sense of belonging, hereby positively impacting mental and social well-being, which is a crucial part of health according to the WHO definition [12];
- These folk responses need to be urgently evaluated within the context of the pandemic, not only for their possible bioactivity and pharmacological properties (as immunomodulators, anti-flu, and cough-relieving agents), but foremost for their diverse cultural meanings and importance;
- In order to maintain resilience in times of crises, communities need to stay properly informed by government agencies, their public spokespeople, and the media, who should collaborate closely with the scientific community to communicate new information about these food medicines in clear, evidence-based, and unambiguous ways.

## Conclusion

This perspective paper underscores that *food medicines* are an essential tool for communities to maintain their holistic well-being during challenging times. We need to devote more attention to studying these food medicines used in community health systems beyond understanding their alleged biological activities, especially for designing appropriate health strategies and to increase widespread awareness of their cultural importance and observed changes in their actual use.

In order for home remedies to be maintained and even promoted within communities, certain conditions need to be present, including personal experimentation, subjective perception of a remedy’s effectiveness, cultural identification or adherence to ideologies (for example natural and healthy movements), and trust. Trust is an essential component for the promotion of knowledge of medicinal plants and healthy foods, which can be instilled by endorsements from celebrities, social media influencers, or come from other information sources. While the traditional routes of knowledge transmission remain relevant, other pathways, notably new media (TV, Internet, social networks, WhatsApp), are quickly becoming increasingly important sources of knowledge. The trustworthiness and scientific accuracy of those new sources of knowledge transmission of home remedies will be a crucial aspect to address in the future, not only during a major health crisis, but also for minor, infectious, and chronic health issues.

Crises like the current, ongoing pandemic are exceptional, but low technological and easily available solutions for health care that are informed by TEK can assist in envisioning more robust, institutionalized, local, and global health strategies, which have dramatically failed since the start of the pandemic in several areas of the globe. 

## Supplementary Information

**Additional file 1.** Details on the methods and questions used in each case study, and primary and secondary sources of information.

**Additional file 2.** Detailed description of the methodology used for Poland, Belarus and Lithuania.

**Additional file 3.** Overview of plant taxa reported in all case studies by country, region or city.

## References

[1] Pieroni A, Price LL (2006). Eating and healing: traditional food as medicine.

[2] Berkes F (1999). Sacred ecology. Traditional ecological knowledge and resource management.

[3] Etkin NL (2006). Edible medicines. An ethnopharmacology of food.

[4] Bruhn JG, Rivier L (2019). Ethnopharmacology - a journal, a definition and a society. J Ethnopharmacol.

[5] Ang L, Song E, Lee HW, Lee MS (2020). Herbal medicine for the treatment of coronavirus disease 2019 (COVID-19): a systematic review and meta-analysis of randomized controlled trials. J Clin Med.

[6] Panyod S, Ho CT, Sheen LY (2020). Dietary therapy and herbal medicine for COVID-19 prevention: a review and perspective. J Tradit Complement Med..

[7] Dâmaris S, Prieto-Garcia JM, Boylan F, Estrada O, Fonseca-Bazzo YM, Jamal CM (2020). COVID-19: is there evidence for the use of herbal medicines as adjuvant symptomatic therapy?. Front Pharmacol.

[8] Fan Y, Zhang Y, Tariq A, Jiang X, Ahamd Z, et al. Food as medicine: a possible preventive measure against coronavirus disease (COVID-19). Phytother Res. in press. 10.1002/ptr.6770. 10.1002/ptr.6770 PMC7283886 32468635

[9] Vandebroek I, Pieroni A, Stepp JR, Hanazaki N, Ladio A, Alves RRN, et al. Reshaping the future of ethnobiology research after the COVID-19 pandemic. Nat Plants. 2020; 10.1038/s41477-020-0691-6. 10.1038/s41477-020-0691-6 32572213

[10] The Plant List. 2013. http://www.theplantlist.org/. (Accessed 5 Sept 2020).

[11] Stevens PF. Angiosperm Phylogeny Website. Version 14, 2017. http://www.mobot.org/MOBOT/research/APweb/. (Accessed 5 Sept 2020).

[12] World Health Organization. http://who.int/about/who-we-are/constitution. (Accessed 21 Nov 2020).

