# Supplementary material for: Taming the pandemic? The importance of homemade plant-based foods and beverages as community responses to COVID-19
Source: J Ethnobiol Ethnomed. 2020 Dec 9;16:75. doi: 10.1186/s13002-020-00426-9 (PMC7724619; doi:10.1186/s13002-020-00426-9)
Supplement: Supplementary file 2 — Additional file 2. Detailed description of the methodology used for Poland, Belarus and Lithuania. [file 13002_2020_426_MOESM2_ESM.pdf]

Additional file 2. Detailed description of the methodology used for Poland, Belarus and Lithuania.

Non-participant observation is a research technique whereby the researcher observes study participants, with their knowledge, but without taking an active part in the situation under scrutiny.

Poland

All data for Poland were obtained from Facebook and followed its terms and conditions for protecting users. The search was done using a newly created nascent account to avoid bias in the search results. Data was gathered from 123 posts in 6 Polish popular discussion groups on herbal medicine (with 23,550 to 54,696 subscribers) under the common question from subscribers "What do you use to prevent (or) treat the Coronavirus?"

Name	Internet address	Type	Number of subscribers
Zioła i Terapie Naturalne	https://www.facebook.com/groups/leczenie/	Private group	33,881
Zioła i Leczenie Ziołami	https://www.facebook.com/groups/991346677667809/	Private group	54,696
Zioła - Leczenie ziołami _ pytania i porady_zioła w kuchni i dla zdrowia	https://www.facebook.com/groups/314618571963812	Private group	45,126
ZIOŁA I TYLKO O ZIOŁACH I ICH ZASTOSOWANIU	https://www.facebook.com/groups/596666917184080	Private group	30,791
Zioła - Ziołolecznictwo - Zdrowe odżywianie - Porady	https://www.facebook.com/groups/ziola.ziololecznictwo/	Private group	23,550
Człowieku lecz się sam - alternatywne porady zdrowotne	https://www.facebook.com/groups/538230256379551/	Private group	53,110

Lithuania

For Lithuania, 71 posts were analyzed from the most popular platforms for folk discussion on home remedies against the Coronavirus. These represented discussion threads on local news portals and forums. Media outlets for inclusion were selected from major Lithuanian news portals such as delfi.lt, lrytas.lt (topics related to Coronavirus news), TV stations (lrt.lt, tv3.lt, dzukijostv.lt) (topics were related to Coronavirus news, interviews with herbalists).

Belarus

Also for Belarus, popular platforms for folk discussion on home remedies against the Coronavirus were the discussion threads on local news portals such as tut.by, onliner.by and independent news, media and service internet portals (discussion threads of news articles about the Covid-19 pandemic) and forums: talks.by, forum.onliner.by (topics related to information about the spread of the Coronavirus in Belarus). For sharing of information during the pandemic, Telegram channels gained popularity. We gathered information from the three most popular of these channels.

Name	Internet address	Number of subscribers
VIRUS Belarus Live	https://t.me/virusbelarus	47,579
Официальный Минздрав	https://t.me/minzdravbelarus	42,882
Трактор Лечит News	https://t.me/traktorlechit	32,299
